# Interleukin 8 and Pentaxin (C-Reactive Protein) as Potential New Biomarkers of Bovine Tuberculosis

**DOI:** 10.1128/JCM.00274-19

**Published:** 2019-09-24

**Authors:** Xintao Gao, Xiaoyu Guo, Ming Li, Hong Jia, Weidong Lin, Lichun Fang, Yitong Jiang, Hongfei Zhu, Zhifang Zhang, Jiabo Ding, Ting Xin

**Affiliations:** a Institute of Animal Sciences (IAS), Chinese Academy of Agricultural Sciences (CAAS), Beijing, China; b China Institute of Veterinary Drugs Control, Beijing, China; c Biotechnology Research Institute, Chinese Academy of Agricultural Sciences (CAAS), Beijing, China; d Molecular and Cellular Biology, Gembloux Agro-Bio Tech University of Liège (ULg), Gembloux, Belgium; University of Tennessee at Knoxville

**Keywords:** CRP, IL-8, biomarker, bovine tuberculosis, proteomics

## Abstract

Bovine tuberculosis (bTB) is caused by Mycobacterium bovis. During the early stage of infection, greater than 15% of M. bovis-infected cattle shed mycobacteria through nasal secretions, which can be detected by nested PCR. To compare the differences in the protein profiles of M. bovis-infected cattle that were nested PCR positive (bTB_PCR-P_) and M. bovis-infected cattle that were nested PCR negative (bTB_PCR-N_) and to screen for biomarkers that will facilitate the early and accurate detection of bTB, we investigated the protein expression profiles of serum and bovine purified protein derivative (PPD-B)-stimulated plasma among bTB_PCR-P_ (*n* = 20), bTB_PCR-N_ (*n* = 20), and uninfected cattle (NC; *n* = 20) by iTRAQ labeling coupled with two-dimensional liquid chromatography-tandem mass spectrometry (iTRAQ-2D LC-MS/MS). After comprehensive analysis, we selected 15 putative differentially expressed serum proteins and 15 plasma proteins for validation by parallel reaction monitoring (PRM) with the same cohort used in the iTRAQ analysis. Four serum and five PPD-B-stimulated proteins were confirmed in follow-up enzyme-linked immunosorbent assays. PPD-B-stimulated interleukin 8 (IL-8) displayed the potential to differentiate M. bovis-infected cattle from NC, with an area under the curve (AUC) value of 0.9662, while PPD-B-stimulated C-reactive protein (CRP) displayed the potential to differentiate bTB_PCR-P_ from bTB_PCR-N_, with an AUC value of 1.00. Finally, double-blind testing with 244 cattle indicated that the PPD-B-stimulated IL-8 test exhibited good agreement with traditional tests (κ > 0.877) with a >90% relative sensitivity and a >98% relative specificity; the PPD-B-stimulated CRP test displayed good agreement with nested PCR (κ = 0.9117), with an observed 94% relative sensitivity and 97% relative specificity. Therefore, the PPD-B-stimulated IL-8 and CRP tests could be used to detect bTB and to differentiate bTB_PCR-P_ from bTB_PCR-N_.

## Abstract (precis)

Bovine tuberculosis (bTB) is caused by Mycobacterium bovis. During the early stage of infection, greater than 15% of M. bovis-infected cattle shed mycobacteria through nasal secretions, which can be detected by nested PCR.

## INTRODUCTION

Bovine tuberculosis (bTB) is a zoonotic infectious disease primarily caused by Mycobacterium bovis (1, 2). M. bovis is a slow-growing pathogen that may incubate in an infected animal for years before the disease becomes clinically evident (3). M. bovis can infect wild animals and humans via an airborne route, and greater than 15% of M. bovis-infected cattle shed the mycobacteria through nasal secretions, especially during the early stages of infection (4).

Traditional methods of diagnosis of bTB include the tuberculin skin test (TST) and interferon gamma (IFN-γ) release assays (IGRAs). TST, a globally accepted method, is recommended by the World Organization for Animal Health (OIE) as the standard method for bTB diagnosis. IGRA is based on the detection and comparison of cell-mediated responses induced by bovine purified protein derivatives (PPD-B) and avian purified protein derivatives (PPD-A) (5). PPD-A testing is used with TST or IGRA to exclude other environmental *Mycobacterium* infections, but it often fails to detect coinfections of M. bovis and M. avium subsp. *paratuberculosis* in cattle. Research has focused on identifying new M. bovis-specific antigens to facilitate a higher specificity in bTB diagnoses. Recent work demonstrated that the sensitivity and specificity of the CFP-10/ESAT-6/TB10.4-based skin test (CET-ST) were 92.31% and 97.3%, respectively (6–8).

Currently, the control of bTB primarily relies on the test-and-slaughter program. Using this strategy, all cattle shown to be positive by TST and/or complementary IGRAs are slaughtered. However, this approach produces a significant economic burden (9). Insufficient government subsidies and a lack of awareness by farmers regarding bTB have led to challenges in implementing this program. Given these challenges, improved diagnostic methods are expected to facilitate control of this disease and reduce the economic burden. Considering that TSTs and IGRAs cannot distinguish the state of M. bovis infection, an *mpb70*-based nested PCR assay was established for detecting mycobacteria in milk and colostrum (9). Previous studies demonstrated that 23.18% to 87.5% of the M. bovis bacteria shed in the nasal exudates of infected cattle can be detected using nested PCR. We recommend that farm owners prioritize the slaughter of PCR-positive cattle in order to control the transmission of mycobacteria. However, application of nested PCR to detect excreters can be performed only in highly specialized facilities. Therefore, screening for novel biomarkers that correlate with PCR positivity and the establishment of an early and accurate blood-based test for bTB will aid in controlling this disease and reducing the economic burdens in developing countries.

**FIG 1:**
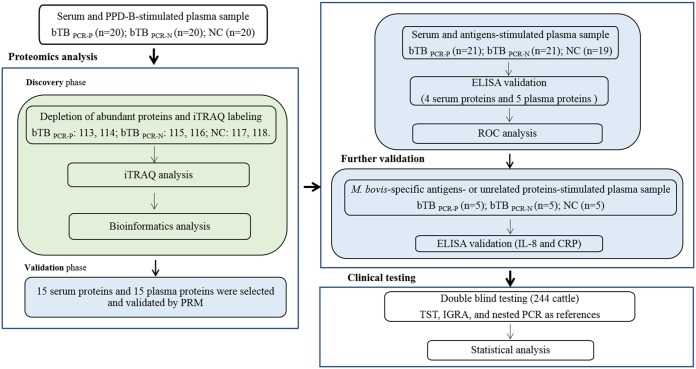
Experimental design work flow. Proteomics of serum and PPD-B-stimulated plasma from PCR-positive M. bovis-infected cattle (bTB_PCR-P_), PCR-negative M. bovis-infected cattle (bTB_PCR-N_), and uninfected cattle (NC) were identified using the iTRAQ-2D LC-MS/MS technology and then validated by parallel reaction monitoring (PRM) and ELISAs.

In this study, we explored new biomarkers of bTB by screening for proteins that are associated with different stages of M. bovis infection. First, cattle were divided into three groups: M. bovis-infected cattle that were nested PCR positive (bTB_PCR-P_), M. bovis-infected cattle that were nested PCR negative (bTB_PCR-N_), and uninfected cattle (NC), as determined by TST, CET-ST, IGRA, a CFP-10–ESAT-6 (CE)-based IGRA, and nested PCR. Proteins in serum or PPD-B-stimulated plasma that were differentially expressed (DE) between these three groups were identified and screened using iTRAQ labeling coupled with two-dimensional liquid chromatography-tandem mass spectrometry (iTRAQ-2D LC-MS/MS). Second, a total of 15 serum proteins and 15 plasma proteins were selected and validated using parallel reaction monitoring (PRM)-based quantitation. Third, four serum proteins (serum amyloid A protein [SAA], alpha-1-acid glycoprotein [AGP], serotransferrin [TF], and alpha-2-HS-glycoprotein [AHSG]) and five PPD-B-stimulated plasma proteins (interleukin 8 [IL-8], TF, pentaxin [C-reactive protein {CRP}], AGP, and complement component C6 [C6]) were further confirmed by enzyme-linked immunosorbent assay (ELISA). The accuracy of new potential biomarkers (PPD-B-stimulated IL-8 and CRP) to differentiate bTB_PCR-P_ and bTB_PCR-N_ or infected cattle and NC was analyzed using receiver operating characteristic (ROC) curves. Finally, the efficiency of the new biomarkers was assessed in a total of 244 cattle, using TST, IGRA, and nested PCR as references (the work flow is shown in Fig. 1).

## MATERIALS AND METHODS

### Animals and sample collection.

bTB in Holstein cattle (Exact 1211) was detected by TST and CET-ST (6). Heparinized whole blood was collected from each cow and dispensed into 24-well tissue culture trays (1.5 ml/well, four wells per animal). The blood samples were stimulated with 0.1 ml PPD-B (300 mg/ml; Prionics AG, Schlieren, Switzerland), PPD-A (300 mg/ml; Prionics AG, Schlieren, Switzerland), phosphate-buffered saline (PBS), or CFP-10–ESAT-6 (CE; 20 μg/ml; expressed and purified in IAS-CAAS with a Trx-His-S tag at the N terminus and with an endotoxin concentration of <10 endotoxin units [EU]/mg) in a 24-well tissue culture tray. Plasma was collected from each well after incubation with antigens for 20 to 24 h at 37°C in 5% CO_2_. The collected plasma was used for IGRA and CE-based IGRA, as summarized in a previous study (6). Blood samples were collected and clotted in vacuum tubes without anticoagulant and then centrifuged at 1,500 × *g* at 4°C for 15 min to collect the serum. The serum collected from each animal was used for M. avium subsp. paratuberculosis and brucellosis antibody tests. All samples were aliquoted in sterile microcentrifuge tubes and frozen at −80°C until further use. Nasal swab specimens were collected from cattle infected with M. bovis, and infection was further confirmed by nested PCR, as previously reported (7).

### Protein extraction and labeling with iTRAQ reagents.

One aliquot of each serum or PPD-B-stimulated plasma sample was thawed on ice and centrifuged at 12,000 rpm at 4°C for 5 min, and the supernatant was collected. We mixed equal amounts of 10 different serum/plasma samples to produce pooled samples in order to increase the accuracy and precision of our proteomics data. Twenty samples were randomly divided into two pools as biological replicates to ultimately generate six iTRAQ-labeled serum pools and six plasma pools.

High-abundance serum/plasma proteins, such as albumin, IgG, and haptoglobin, were removed using a ProteoMiner protein enrichment kit (Bio-Rad, USA). After the removal of high-abundance serum/plasma proteins, the samples were reduced, digested, and labeled in turn. First, samples containing proteins in the elution buffer were reduced by incubating with 10 mM dithiothreitol (DTT) at 56°C for 45 min and then alkylated by incubating with 55 mM iodoacetamide for 1 h in the dark. Samples were then precipitated with two times the volume of ice-cold acetone at –20°C for 2 h. After centrifugation, protein pellets were air dried and dissolved in 0.2 ml of triethyl ammonium bicarbonate buffer (TEAB; 100 mM; pH 8.0). The total protein concentration was determined by a Bradford protein assay (Quick Start Bradford protein assay kit; Bio-Rad, USA). Second, 100 μg of protein from each group was digested with trypsin (at a trypsin/sample [wt/vol] ratio of 1:50) at 37°C for 12 to 16 h. The digested peptides were desalted with a Strata-X C_18_ column (Waters, Milford, MA, USA) and dried using vacuum centrifugation. The dried peptide powder was dissolved in 20 μl of TEAB (0.5 M). The digested samples were further labeled with iTRAQ reagents (AB Sciex U.K. Limited) in sequence, as follows: bTB_PCR-P_, iTRAQ tags 113 and 114; bTB_PCR-N_, iTRAQ tags 115 and 116; NC, iTRAQ tags 117 and 118. All labeled serum or plasma samples were pooled in equal amounts. The labeled serum or plasma samples were fractionated using high-performance liquid chromatography (HPLC; Thermo Dinoex Ultimate 3000 BioRS system) using a Durashell C_18_ (particle size, 5 μm; 100 Å; 4.6 by 250 mm) column. A total of 16 fractions were collected.

### LC-MS/MS analysis.

LC-MS/MS analysis was performed on a TripleTOF 5600+ mass spectrometer coupled with an Eksigent nanoLC system (AB Sciex, USA). The peptides were loaded onto a C_18_ trap column (particle size, 5 μm; 100 μm by 20 mm) and eluted at 300 nl/min onto a C_18_ analytical column (particle size, 3 μm; 75 μm by 150 mm) over a 90-min gradient. The two mobile phases were buffer A (0.1% [vol/vol] formic acid, 5% [vol/vol] acetonitrile) and buffer B (0.1% [vol/vol] formic acid, 95% [vol/vol] acetonitrile).

### Protein identification and relative quantification.

The original MS/MS data were analyzed using ProteinPilot software (v4.5). MS/MS data were searched against the Bos taurus UniProt database (containing 32,195 sequences). Parameters were set as follows: TripleTOF 5600+ instrument, iTRAQ quantification, and cysteine modified with iodoacetamide. The biological modifications selected were ID focus, trypsin digestion, and quantitate. Bias correction and background correction were selected for protein quantification and normalization. For false discovery rate (FDR) calculation, an automatic decoy database search strategy was used to estimate FDR using the PSPEP (Proteomics System Performance Evaluation Pipeline software) algorithm. Only proteins with at least one unique peptide and an unused score of >1.3 were considered for further analysis. Proteins with expression ratios of >1.5-fold or <0.67-fold and with *P* values of <0.05 were considered significant.

### Bioinformatics analysis.

The functional annotations of proteins, including their cellular component, molecular function, and biological process, were analyzed using the Gene Ontology (GO) database (http://geneontology.org/). Pathway analysis of DE proteins was conducted using the Kyoto Encyclopedia of Genes and Genomes (KEGG) database.

### PRM-based quantification.

iTRAQ results were validated by targeted MS analysis using parallel reaction monitoring (PRM), performed on a TripleTOF 5600+ LC-MS/MS system (AB Sciex). Proteins were prepared in a manner similar to that used to prepare the iTRAQ-labeled samples. MS data acquisition was first performed in the data-dependent acquisition (DDA) mode to obtain MS/MS spectra for the 40 most abundant precursor ions following each survey MS1 scan in each cycle. Protein Pilot software was used to identify proteins, and spectrum library building was accomplished by importing the database search results into Skyline software. Target proteins for PRM validation were imported into Skyline software, and the peptides for protein quantification were selected according to the ion signals in the spectrum library. A list of associated peptides containing *m/z* values and retention times was exported from Skyline software and then imported into Analyst MS control software for PRM acquisition method construction. Data collection from each sample was performed using the final PRM acquisition method on a QqTOF mass spectrometer, where each precursor ion was selected by the quadrupole and fragmented and then all fragment ions were quantified in the time of flight (TOF) mass analyzer. To eliminate protein carryover, a blank was run between adjacent samples to wash the column. Data processing was performed using Skyline software, and the quantification results were manually inspected for each peptide of the targeted proteins and normalized by the total peak area.

### ELISAs.

Candidate biomarkers for bTB were validated using a bovine IL-8 ELISA kit (Mabtech Inc., Cincinnati, OH, USA), a bovine SAA1 ELISA kit (Aviva Systems Biology, San Diego, CA, USA), a bovine CRP ELISA kit (Aviva Systems Biology, San Diego, CA, USA), a bovine transferrin ELISA kit (Aviva Systems Biology, San Diego, CA, USA), a bovine IP-10 ELISA VetSet kit (Kingfisher Biotech Inc., Saint Paul, MN, USA), and a bovine IL-17A ELISA VetSet kit (Kingfisher Biotech Inc., Saint Paul, MN, USA). Protein levels in serum or plasma were detected according to the manufacturers’ instructions.

To determine the cutoff values of the PPD-B-stimulated IL-8, CRP, IP-10, or IL-17A ELISAs, PPD-B-stimulated plasma from 19 healthy cattle (from a tuberculosis [TB]-free herd negative by TST, CET-ST, IGRA, CE-based IGRA, and PCR analysis), 21 bTB_PCR-P_ (positive by TST, CET-ST, IGRA, CE-based IGRA, and PCR analysis), and 21 bTB_PCR-N_ (positive by TST, CET-ST, IGRA, and CE-based IGRA but negative by PCR analysis) were prepared as described above. The levels of IL-8, CRP, IP-10, and IL-17A were detected using ELISA kits. Differences in the concentrations of each of the cytokines were analyzed by receiver operating characteristic (ROC) analysis (GraphPad Prism [version 5] software).

### IL-8 and CRP response specificity in M. bovis-infected cattle.

To assess the specificity of the IL-8 and CRP responses in M. bovis-infected cattle, bTB_PCR-P_ (*n* = 5), bTB_PCR-N_ (*n* = 5), and NC (*n* = 5) were identified by TST, IGRA, CE-based IGRA, and nested PCR. Heparinized whole blood was collected from each cow and dispensed into 24-well tissue culture trays (1.5 ml/well, five wells per animal). Blood samples were stimulated with 0.1 ml of PPD-B (3,000 IU/ml; Prionics AG, Schlieren, Switzerland), CE (20 μg/ml), PET (Trx-His-S tag protein with an endotoxin concentration of <10 EU/mg), bovine serum albumin (BSA; 20 μg/ml, Sigma), or PBS in 24-well tissue culture trays. Plasma was collected as mentioned above. The concentrations of IL-8 and CRP were tested using ELISA kits.

### PPD-B-stimulated IL-8 and CRP tests used in clinical testing.

To evaluate the efficiency of the PPD-B-stimulated IL-8 and CRP tests, a total of 244 cattle (42 males and 202 females, including the data used for ROC analysis) from four dairy farms were double-blind tested using TST, IGRA, nested PCR, and PPD-B-stimulated IL-8 and CRP tests.

### Statistical analysis.

Data were analyzed by analysis of variance (ANOVA) followed by a Kruskal-Wallis or Spearman correlation test using GraphPad Prism (version 5) software (San Diego, CA). A *P* value (two-tailed) of <0.05 was considered significant. Agreement between tests was evaluated using the κ coefficient.

## RESULTS

### Sample collection.

Based on an initial screening of serum proteins related to M. bovis infection, 82 naturally M. bovis-infected cattle and 39 NC were identified by TST, CET-ST, IGRA, and CE-based IGRA. In addition, M. bovis-infected cattle were subdivided into the bTB_PCR-P_ (*n* = 41) and bTB_PCR-N_ (*n* = 41) groups using nested PCR. Of these samples, 20 bTB_PCR-P_ and 20 bTB_PCR-N_ and 20 NC were utilized for iTRAQ and PRM analyses, and the remaining 21 bTB_PCR-P_, 21 bTB_PCR-N_, and 19 NC were used for ELISA validation.

### Identification and relative quantification of serum proteins.

We compared three groups of mixed serum samples with two biological replicates in each group (i.e., the bTB_PCR-P_ group [labeled with iTRAQ tags 113 and 114], the bTB_PCR-N_ group [labeled with iTRAQ tags 115 and 116], and the NC group [labeled with iTRAQ tags 117 and 118]), using iTRAQ-2D LC-MS/MS analysis. We identified a total of 845 proteins; of these, 681 had two or more peptides. A total of 223 proteins were found to be significantly DE (fold change, ≥1.5 or ≤0.67; *P* ≤ 0.05) between M. bovis-infected cattle (bTB_PCR-P_ and bTB_PCR-N_) and NC (*n* = 20), including 112 upregulated proteins and 111 downregulated proteins (see Table S1 in the supplemental material). A total of 74 DE proteins, including 46 upregulated and 28 downregulated proteins (Table S1), were identified between bTB_PCR-P_ and bTB_PCR-N_. Gene ontology analysis indicated that most of the identified DE proteins are involved in cellular processes, biological regulation, and the response to stimuli; are located in the extracellular region and organelles; and possess binding, catalytic, and enzyme regulator activities (Table S1). By KEGG analysis, the largest proportion of these proteins was associated with complement and coagulation cascades (Fig. S1A-1 and A-2). Venn diagram analysis revealed 35 DE proteins shared among the bTB_PCR-P_, bTB_PCR-N_, and NC groups (Fig. S1B-1 and Table S2). Based on the bioinformatic and Venn diagram analyses, coupled with the fold changes in protein expression, 15 proteins were selected for preliminary verification (Table S3).

### Identification and relative quantification of PPD-B-stimulated plasma proteins.

Although all animals in this study were free from paratuberculosis and brucellosis and their infections were confirmed by TST, IGRA, and PCR, it is difficult to exclude the possibility of infection with additional pathogens. To screen for proteins in the blood that are associated with M. bovis infection, PPD-B-stimulated plasma from M. bovis-infected cattle and NC were compared by iTRAQ-2D LC-MS/MS analysis. We identified 719 proteins; of these, 531 proteins had two or more peptides. In total, we identified 207 DE proteins (fold change, ≥1.5 or ≤0.67; *P *≤ 0.05), including 151 upregulated and 56 downregulated proteins, between M. bovis-infected cattle (bTB_PCR-P_ and bTB_PCR-N_) and NC (Table S4). A total of 107 DE proteins, including 75 upregulated and 32 downregulated proteins, were identified between bTB_PCR-P_ and bTB_PCR-N_ (Table S4). Gene ontology analyses indicated that the DE proteins enriched in PPD-B-stimulated plasma have biological processes, cellular components, and molecular functions similar to those of the DE proteins identified to be enriched in serum (Table S4). Similarly, KEGG analysis revealed that the largest proportion of those proteins was also associated with complement and coagulation cascades (Fig. S1C-1 and C-2). We observed 37 DE proteins shared between the bTB_PCR-P_, bTB_PCR-N_, and NC groups by Venn diagram analysis (Fig. S1B-2 and Table S2). Based on the bioinformatics and Venn diagram analyses coupled with the fold changes, 15 were selected for further verification (Table S3).

### Validation of iTRAQ analysis results by PRM.

A total of 15 serum proteins and 15 PPD-B-stimulated proteins from the bTB_PCR-P_, bTB_PCR-N_, and NC groups were selected and subjected to PRM-based relative quantification (Table S5). The relative abundances of peptides from those individual proteins were acquired and normalized by the corresponding total peak area. By PRM, eight proteins in serum (AHSG, CGN1, OMD, SAA, C4, C7, APOE, and TF) and nine proteins in PPD-B-stimulated plasma (TF, IL-8, CD14, LRG1, C6, AGP, EFEMP2, AMBP, and RSU1) exhibited fold changes in expression similar to those identified by iTRAQ (Table S3). Although similarly pooled samples were used to conduct the iTRAQ and PRM analyses, the PRM results were not completely consistent with those obtained by iTRAQ. Beta-2-microglobulin and vitamin D-binding protein (serum), as well as PPD IL1RN, MBL, OMD, and protein HP-25 homolog 1 (PPD-B-stimulated plasma), were not detected by PRM (Table S3). Furthermore, five proteins in serum (AGP, AMBP, asporin, SERPINA1, and C6) and CRP in plasma exhibited contrasting results between PRM and iTRAQ analyses (Table S3). The removal of high-abundance proteins from serum and plasma samples may affect the detection of other low-abundance proteins. Considering our iTRAQ and PRM results, we further validated the levels of four serum proteins (AGP, AHSG, SAA, and TF) and five plasma proteins (CRP, TF, IL-8, C6, and AGP) using commercial ELISA kits.

### Validation of potential bTB biomarkers by ELISA.

To assess whether these four serum proteins (AGP, AHSG, SAA, and TF) and five plasma proteins (CRP, TF, IL-8, C6, and AGP) could be used to detect M. bovis infection and determine the stage of M. bovis infection, serum and antigen-stimulated plasma was harvested from 42 M. bovis-infected cattle (including 21 bTB_PCR-P_ and 21 bTB_PCR-N_) and 19 NC, and the proteins were detected using ELISA kits.

**FIG 2:**
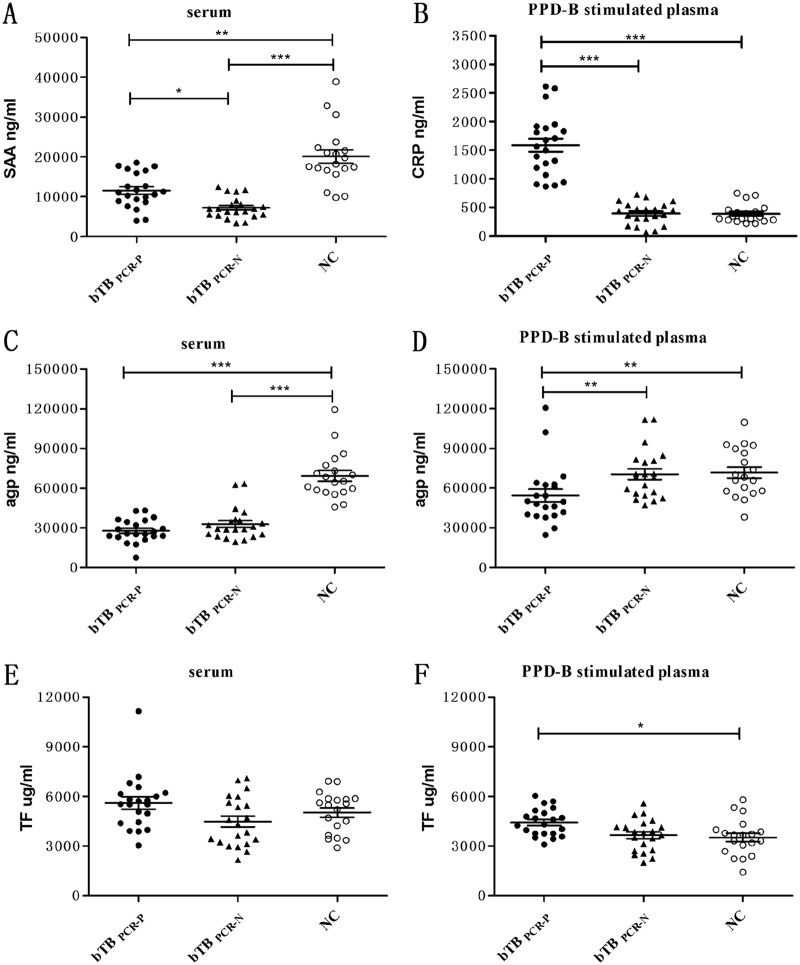
Levels of SAA, CRP, AGP, and TF in bTB_PCR-P_, bTB_PCR-N_, and NC. (A) SAA levels in serum. (B) CRP levels in PPD-B-stimulated plasma. (C) AGP levels in serum. (D) AGP levels in PPD-B-stimulated plasma. (E) TF levels in serum. (F) TF levels in PPD-B-stimulated plasma. Whole blood was collected from 21 bTB_PCR-P_, 21 bTB_PCR-N_, and 19 NC and stimulated with PPD-B for 24 h. Serum and plasma were harvested to measure the levels of SAA, CRP, AGP, and TF using commercial kits. Significant differences in protein levels between bTB_PCR-P_, bTB_PCR-N_, and NC were determined using a one-way ANOVA (Kruskal-Wallis test) followed by Dunn’s multiple-comparison test. *, *P *< 0.05; **, *P *< 0.001; ***, *P *< 0.0001.

The concentrations of SAA in the serum of M. bovis-infected cattle (including bTB_PCR-P_ and bTB_PCR-N_) were significantly less than those in NC. SAA levels were significantly higher in bTB_PCR-P_ than in bTB_PCR-N_ (Fig. 2A). CRP concentrations in PPD-B-stimulated plasma were significantly higher in bTB_PCR-P_ than in bTB_PCR-N_ and NC (Fig. 2B). Serum AGP levels were significantly higher in NC than in M. bovis-infected cattle. However, following PPD-B stimulation, AGP levels increased in M. bovis-infected cattle, and the AGP level was significantly higher in bTB_PCR-P_ than in bTB_PCR-N_ (Fig. 2C and D). For serum TF levels, no significant differences were observed between M. bovis-infected cattle and NC. TF levels decreased following stimulation with PPD-B, and TF was significantly higher in bTB_PCR-P_ than in NC (Fig. 2E and F). The concentrations of AHSG (serum) and C6 (plasma) were similar in M. bovis-infected cattle and NC (data not shown).

**FIG 3:**
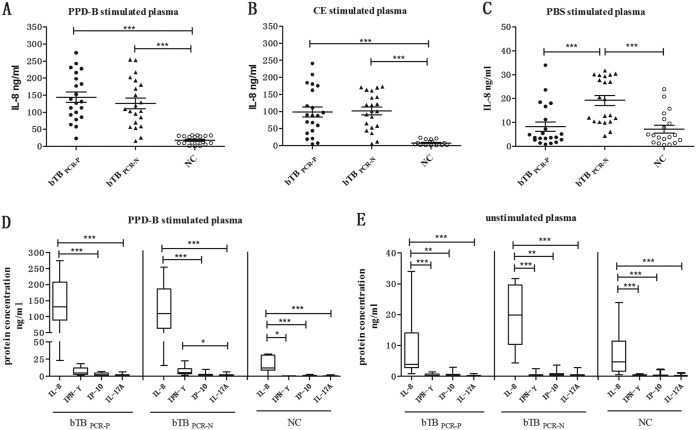
Cytokine levels in bTB_PCR-P_, bTB_PCR-N_, and NC. (A) IL-8 induced by PPD-B. (B) IL-8 induced by CE. (C) IL-8 induced by PBS. (D) Concentrations of IL-8, IFN-γ, IP-10, and IL-17A induced by PPD-B. (E) Concentrations of IL-8, IFN-γ, IP-10, and IL-17A induced by PBS. Whole blood was obtained from 21 bTB_PCR-P_, 21 bTB_PCR-N_, and 19 NC and then stimulated with PPD-B, CE, or PBS for 24 h. Serum and plasma were harvested to measure cytokine levels using commercial kits. Significant differences in protein concentrations were determined using a one-way ANOVA (Kruskal-Wallis test) followed by Dunn’s multiple-comparison test. *, *P *< 0.05; **, *P *< 0.001; ***, *P *< 0.0001.

PPD-B- or CE-induced IL-8 levels were increased in bTB_PCR-P_ and bTB_PCR-N_ and were significantly higher than those in NC. However, the levels of unstimulated IL-8 (treated with PBS) were significantly higher in bTB_PCR-N_ than in bTB_PCR-P_ and NC (Fig. 3A to C). To further analyze the diagnostic potential of IL-8 for bTB, the correlations among IL-8, our previously published potential biomarkers (IL-17A, IP-10), and PPD-B-induced IFN-γ, CE, or PBS were calculated. This comparison included all individuals and all treatments (*n* = 61 [21 bTB_PCR-P_ + 21 bTB_PCR-N_ + 19 NC]). As shown in Table 1, PPD-B- or CE-induced IL-8, IL-17A, IP-10, and IFN-γ levels showed significant correlations with one another, and the IL-8 level was moderately correlated with the IFN-γ level. The PBS-induced IL-8 level exhibited a weak correlation with the IP-10 and IL-17 levels but no correlation with the IFN-γ level. Moreover, IL-8 concentrations in PPD-B-stimulated or unstimulated plasma were significantly greater than those of IFN-γ, IP-10, or IL-17A (Fig. 3D and E). These data indicate that PPD-B-stimulated IL-8, IP-10, and IL-17A have the potential to differentiate M. bovis-infected cattle from NC, while PPD-B-stimulated CRP, PPD-B-stimulated AGP, unstimulated IL-8, and serum SAA could potentially differentiate bTB_PCR-P_ from bTB_PCR-N_.

**TABLE 1:** Correlations between IL-8, IFN-γ, IP-10, and IL-17A induced by PPD-B or CE^*a*^

Protein	Correlation coefficient (Spearman’s *r*)							
	PPD-B-stimulated plasma				CE-stimulated plasma			
	IL-8	IFN-γ	IP-10	IL-17A	IL-8	IFN-γ	IP-10	IL-17A
IL-8		0.75*	0.63*	0.54*		0.63*	0.53*	0.48*
IFN-γ	0.75*		0.63*	0.61*	0.63*		0.62*	0.52*
IP-10	0.63*	0.63*		0.47*	0.53*	0.62*		0.44*
IL-17A	0.54*	0.61*	0.47*		0.48*	0.52*	0.44*	

a Correlation coefficients were determined using Spearman’s two-tailed correlation test. *, *P *< 0.001, significant correlation. Comparisons included all individuals and all treatments (*n* = 61 [21 bTB_PCR-_*_P_* + 21 bTB_PCR-N_ + 19 NC]). bTB_PCR-P_, 21 cattle from a bTB-prevalent dairy farm positive for bTB by TST, CET-ST, IGRA, CE-based IGRA, and nested PCR; bTB_PCR-N_, 21 cattle from a bTB-prevalent dairy farm positive for bTB by TST, CET-ST, IGRA, and CE-based IGRA but negative by nested PCR; NC, 19 cattle from a bTB-free dairy farm determined to be M. bovis negative by TST, CET-ST, IGRA, CE-based IGRA, and nested PCR. Whole blood was collected and stimulated with PPD-B or CE for 24 h. Plasma was harvested to measure the levels of cytokines using commercial kits.

### ROC analysis.

ROC analysis was used to evaluate the diagnostic value of our proteins of interest. The area under the curve (AUC) indicated that PPD-B-stimulated IL-8 displayed a diagnostic ability superior to that of IP-10 and IL-17A in discriminating between M. bovis-infected cattle and NC. PPD-B-stimulated CRP showed a diagnostic value superior to that of PPD-B-stimulated AGP, unstimulated IL-8, and serum SAA in discriminating between bTB_PCR-P_ and bTB_PCR-N_ cattle. To reach the target test specificity of 100%, the cutoff values for the PPD-B-stimulated IL-8 and CRP tests should be set at 43.05 ng/ml and 794.8 ng/ml, respectively (as shown in Table 2 and Fig. S2).

**TABLE 2:** Sensitivity and specificity of cytokine tests estimated by ROC analysis^*a*^

Effect	Diagnostic antigen	Area under ROC curve	Sensitivity (%)	95% CI (%) for sensitivity	Specificity (%)	95% CI (%) for specificity	Cutoff value (ng/ml)
Differentiate M. bovis-infected cattle from NC	**PPD-B-stimulated IL-8**	**0.9662**	92.86	80.52–98.50	94.74	73.97–99.87	>32.57
			**92.86**	**80.52–98.50**	**100**	**82.35–100.0**	**>43.05**
	CE-stimulated IL-8	0.9561	85.71	71.46–94.57	94.74	73.97–99.87	>21.9
			85.71	71.46–94.57	100.0	82.35–100.0	>29.42
	PPD-B-stimulated IP-10	0.9500	52.38	36.42–68.00	95	75.13–99.87	>1.992
			52.38	36.42–68.00	100	83.16–100.0	>2.045
	PPD-B-stimulated IL-17A	0.8464	28.57	15.72–44.58	95	75.13–99.87	>1.767
			28.57	15.72–44.58	100	83.16–100.0	>2.061
Differentiate bTB_PCR-P_ and bTB_PCR-N_	**PPD-B-stimulated CRP**	**1.000**	100	83.89–100.0	95.24	76.18–99.88	>701.3
			**100**	**83.89–100.0**	**100**	**83.89–100.0**	**>794.8**
	Unstimulated IL-8	0.8322	52.38	29.78–74.29	100.0	83.89–100.0	<4.088
			52.38	29.78–74.29	95.24	76.18–99.88	<4.648
	PPD-B-stimulated AGP	0.7732	52.38	29.78–74.29	95.24	76.18–99.88	<49,925
			42.86	21.82–65.98	100.0	83.89–100.0	<46,732
	Serum SAA	0.7914	38.10	18.11–61.56	95.24	76.18–99.88	>12,082
			38.10	18.11–61.56	100.0	83.89–100.0	>12,495

a The cutoff values for the PPD-B-stimulated IL-8, IP-10, and IL-17A ELISAs were obtained using 19 healthy cattle and 42 M. bovis-infected cattle. The cutoff values for the PPD-B-stimulated CRP, PPD-B-stimulated AGP, unstimulated IL-8, and serum SAA ELISAs were obtained using 21 bTB_PCR-P_ and 21 bTB_PCR-N_. CI, confidence interval. The PPD-B-stimulated IL-8 ELISA showed a higher area under the ROC curve (AUC = 0.9662) and was better than the CE-stimulated IL-8, PPD-B-stimulated IP-10, and IL-17A ELISAs at differentiating M. bovis-infected cattle from NC. The PPD-B-stimulated CRP ELISA was better than the unstimulated IL-8, PPD-B-stimulated AGP, and serum SAA ELISAs at differentiating bTBPCR-P and bTBPCR-N. The specificity for each diagnostic antigen was set at nearly 95% or 100%, and the sensitivity, area under the ROC curve, and cutoff value were calculated and are shown.

### Specificity of IL-8 and CRP responses in M. bovis-infected cattle.

**FIG 4:**
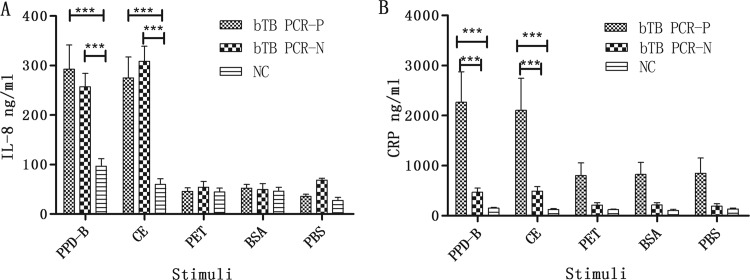
Levels of IL-8 and CRP induced by proteins unrelated to M. bovis in bTB_PCR-P_, bTB_PCR-N_, and NC. (A) IL-8 induced by different stimuli. (B) CRP induced by different stimuli. Samples were obtained from five bTB_PCR-P_, five bTB_PCR-N_, and five NC. Whole blood was collected and stimulated with PPD-B, CE, PET, BSA, or PBS for 24 h. Plasma was harvested to measure cytokine levels using commercial kits. Significant differences between protein concentrations were determined using a two-way ANOVA followed by Bonferroni posttests. ***, *P* < 0.0001.

To access the specificity of the IL-8 and CRP responses in M. bovis-infected cattle, we stimulated whole blood from infected and uninfected cattle with PPD-B, CE, and antigens unrelated to M. bovis. We found that the unrelated antigens and PBS induced similar levels of IL-8 or CRP in infected and uninfected cattle. The levels of PPD-B- or CE-induced IL-8 were significantly higher in M. bovis-infected cattle than in NC, and the levels of PPD-B- or CE-induced CRP were significantly higher in bTB_PCR-P_ than in bTB_PCR-N_ or NC (Fig. 4).

### Clinical testing.

To assess the application of PPD-B-stimulated IL-8 and CRP tests to the diagnosis of bTB, 244 cattle were double-blind tested using TST, IGRA, nested PCR, and PPD-B-stimulated IL-8 and CRP tests. Strong correlations were observed between the concentrations of PPD-B-stimulated proteins (for IL-8 versus IFN-γ, Spearman *r *= 0.75 and *P *< 0.001; for CRP versus IFN-γ, Spearman *r *= 0.339 and *P *< 0.001; for CRP versus IL-8, Spearman *r *= 0.32 and *P *< 0.001). We also observed strong correlations between the increased skin thickness observed by TST and protein concentrations (for IL-8 versus TST, Spearman *r *= 0.81 and *P *< 0.001; for CRP versus TST, Spearman *r *= 0.3 and *P *< 0.001; for IFN-γ versus TST, Spearman *r *= 0.71 and *P *< 0.001). The PPD-B-stimulated IL-8 test exhibited agreement with traditional tests in discriminating between infected and uninfected cattle (for IL-8 versus TST, κ = 0.877; for IL-8 versus IGRA, κ = 0.926). Compared to TST and IGRA, the PPD-B-stimulated IL-8 test had a relative sensitivity and specificity greater than 90% and 98%, respectively (Table 3). When the PPD-B-stimulated CRP test was used to discriminate between bTB_PCR-P_ and bTB_PCR-N_ among 115 M. bovis-infected cattle (the same as those used for the PPD-B-stimulated IL-8 test), it exhibited good agreement with nested PCR (κ = 0.9117). Compared with nested PCR, the relative sensitivity and specificity of the PPD-B-stimulated CRP test were 94% and 97%, respectively (Table 4). Taken together, our clinical testing demonstrated that the PPD-B-stimulated IL-8 and CRP tests can be used to detect bTB and to distinguish bTB_PCR-P_ from bTB_PCR-N_, respectively.

**TABLE 3:** Numbers of tested cattle and agreement between the PPD-B-stimulated IL-8 test and TST or IGRA^*a*^

PPD-B-stimulated IL-8 test result	No. of cattle with the indicated result by:					
	TST			IGRA		
	Positive	Negative	Total	Positive	Negative	Total
Positive	113	2	115	113	2	115
Negative	13	116	129	7	122	129
						
Total	126	118	244	120	124	244

a Cattle from four different farms were tested. The cutoff value for the PPD-B-stimulated IL-8 test was set at 43.05 ng/ml. If the concentration of PPD-B-stimulated IL-8 was ≥43.05 ng/ml, the animal was considered infected with M. bovis. If the concentration of PPD-B-stimulated IL-8 was <43.05 ng/ml, the animal was considered bTB free. κ values were as follows: for the PPD-B-stimulated IL-8 test and TST, κ was 0.877; for the PPD-B-stimulated IL-8 test and IGRA, κ was 0.926. The relative sensitivities of the PPD-B-stimulated IL-8 test compared to the results of TST and IGRA were 90% and 94%, respectively. The relative specificities of the PPD-B-stimulated IL-8 test compared to the results of TST and IGRA were both 98%.

**TABLE 4:** Numbers of cattle tested and agreement between PPF-B-stimulated CRP test and nested PCR^*a*^

PPD-B-stimulated CRP test	No. of cattle with the following nested PCR result:		
	Positive	Negative	Total
Positive	48	2	50
Negative	3	62	65
			
Total	51	64	115

a A total of 115 M. bovis-infected cattle were confirmed to be infected by the PPD-B-stimulated IL-8 test. The cutoff value for the PPD-B-stimulated CRP test was set at 794.8 ng/ml. If the concentration of PPD-B-stimulated CRP was ≥794.8 ng/ml, the animal was considered bTB_PCR-P_. If the concentration of PPD-B-stimulated IL-8 was <794.8 ng/ml, the animal was considered bTB_PCR-N_. For the PPD-B-stimulated CRP test and nested PCR, κ was 0.9117. The relative sensitivity and specificity of the PPD-B-stimulated CRP test compared to the results of nested PCR were 94% and 97%, respectively.

## DISCUSSION

### IL-8 as a promising biomarker for detection of bTB.

Effective control of bTB is reliant on early and accurate diagnosis. However, the established TST and IGRA require 48 to 72 h for a diagnosis and are unable to distinguish the stages of M. bovis infection. Several studies have identified various novel bTB biomarkers. PPD-B-stimulated IL-17A and IP-10 were found to be related to M. bovis infection, and increased PPD-B-induced IL-17A transcripts in peripheral blood mononuclear cells (PBMC) are associated with pathology in M. bovis-infected cattle (10–13). However, a recent study found that PPD-B-induced IP-10 and IL-17A levels are lower than those of IFN-γ and that CE-induced IL-17A levels were similar in bTB_PCR-P_ and NC (14). Our study found that, with a target specificity of 95%, the sensitivities of IP-10 and IL-17A were only 52.38% and 28.57%, respectively. Thus, IP-10 and IL-17A are not suitable for bTB diagnosis, but IP-10 is considered a promising biomarker for human TB and can detect TB in children, HIV-positive patients, and those undergoing therapy for tuberculosis (10, 15–17). Using iTRAQ analysis, Seth et al. confirmed that serum alpha-1-microglobulin/bikunin precursor (AMBP) protein, alpha-1-acid glycoprotein (AGP), fetuin, and alpha-1B glycoprotein levels were significantly increased in M. bovis-infected cattle, and dot analysis revealed a significant increase in the vitamin D-binding protein precursor (DBP) in *Mycobacterium*-infected cattle, but the study lacked validation by ELISA (18). The combined application of untargeted proteomics (iTRAQ, data-dependent acquisition [DDA], and data-independent acquisition [DIA]) and targeted proteomics (PRM and selected/multiple reaction monitoring [SRM/MRM]) to screen and validate biomarkers has been reported in several studies (19–21). This approach dramatically improved the identification of several putative biomarkers in the discovery phase and simplified the work flow using relatively high-throughput validation. These advantages make this combined strategy a powerful tool for the characterization of biomarkers in a wide range of infectious diseases.

The aim of the present study was to identify biomarkers of bTB and to assess differences between bTB_PCR-P_ and bTB_PCR-N_. Serum biomarkers facilitate testing but may not be specific for bTB. Thus, we compared both serum and PPD-B-stimulated plasma proteomes in M. bovis-infected cattle and NC using iTRAQ analysis and validated our results using relatively high-throughput PRM and commercial ELISA kits. We identified a total of 845 serum proteins and 719 plasma proteins, significantly more than were identified in previous studies (18). However, cytokines related to M. bovis infection, such as IFN-γ and IL-17A, were not previously identified by iTRAQ analysis (18). Interestingly, in our study, we identified differences in PPD-B-stimulated IL-8 levels between M. bovis-infected cattle and NC by both iTRAQ and PRM analyses. PPD-B- and CE-stimulated IL-8 levels were significantly increased in M. bovis-infected cattle compared to NC, while unstimulated IL-8 levels were significantly higher in bTB_PCR-N_ than in bTB_PCR-P_ and NC. Furthermore, PPD-B- and CE-induced IL-8 levels exhibited a good correlation with IFN-γ levels, and the concentration of IL-8 was higher than that of IFN-γ, IP-10, or IL-17A. Our results suggest that PPD-B- or CE-stimulated IL-8 could be used to differentiate between M. bovis-infected cattle and NC, while unstimulated IL-8 could differentiate between bTB_PCR-N_ and bTB_PCR-P_. Similarly, another study indicated that CE-stimulated and unstimulated IL-8 levels are significantly higher in active TB patients than latent TB patients and heathy controls, and IL-8 concentrations may help differentiate between active TB and latent M. tuberculosis infection (LTBI) (22). In addition, the levels of IL-8 were higher in patients who died from TB than in survivors; decreased serum IL-8 levels may reflect the status of an infection other than M. tuberculosis infection or infection clearance (23, 24).

Although the exact role of IL-8 in the pathogenesis of TB is not fully understood, recent studies have shown that IL-8 can bind to tubercle bacilli, and the IL-8–pathogen interaction contributes to the increased mycobactericidal properties of macrophages and neutrophils (25). Higher levels of IL-8 contribute to the recruitment of monocytes and T cells to the pulmonary compartment, attract neutrophils to the infection site, and are required for granuloma formation (25, 26). However, TB bacilli avoid the neutrophil response by downregulating IL-8 secretion by infected monocytes through an IL-10-mediated autocrine loop (27). In cattle, although the levels of IL-8 and the role of IL-8 in bTB pathogenesis have been relatively unstudied, our results indicate that IL-8 also plays an important role in bTB. Furthermore, the ROC analysis indicated that PPD-B-stimulated IL-8 is a more suitable biomarker of M. bovis infection than IP-10 or IL-17A. Clinical testing indicated a high relative sensitivity and specificity for the PPD-B-stimulated IL-8 test compared to those of TST or IGRA. Establishing the role of IL-8 as a biomarker for bTB will require verification with a larger cohort of both experimentally and naturally M. bovis-infected cattle and NC.

### PPD-B-stimulated CRP facilitates differentiation of bTB_PCR-P_ and bTB_PCR-N_.

The acute-phase response is a prominent systemic reaction to an organism to local or systemic disturbances in homeostasis due to infection, tissue injury, trauma or surgery, neoplastic growth, or immunological disorders (28). In this study, we identified several positive acute-phase proteins, including SAA, CRP, AGP, complement factors, haptoglobin, and alpha-2-microglobulin, as well as one negative acute-phase protein, TF. Upon validation by ELISA, we found that AGP and SAA levels were significantly decreased in M. bovis-infected cattle and that the serum SAA level was significantly higher in bTB_PCR-P_ than in bTB_PCR-N_. Similarly, the level of serum SAA has been found to be increased in smear-positive compared to smear-negative TB patients (29). However, the variations in the levels of these four acute-phase proteins in TB patients differed from those in M. bovis-infected cattle. In human TB, the SAA level was significantly higher in TB patients than in healthy controls, and it was also significantly increased in TB patients with lung lesions and tended to decline in patients undergoing TB treatment (29–31). The AGP level was found to be increased in active TB patients and may be a possible marker of a slow response to anti-TB treatment (32). Jensen et al. first identified an association between LTBI and elevated AGP levels (33). TF levels in serum and sputum specimens were significantly higher in active TB patients than in latent TB patients (34). It is difficult to explain the higher levels of SAA and AGP in uninfected cattle. Although 39 paratuberculosis- and brucellosis-free NC were randomly selected from two bTB-free dairy farms, the possibility of infection or immunization with other viruses or bacteria, which may lead to elevations in the levels of SAA and AGP, cannot be excluded. Furthermore, infection with M. bovis may inhibit host immune responses, and SAA and AGP levels decreased significantly in M. bovis-infected cattle, but their effect on M. bovis infection requires further investigation. We also detected TF and AGP in PPD-stimulated plasma, and the TF level decreased in both M. bovis-infected cattle and NC following PPD-B stimulation, while the AGP level was increased in M. bovis-infected cattle. PPD-B may activate acute-phase proteins, leading to increased AGP and decreased TF. As variations in serum SAA and AGP are not specific to M. bovis infection, they are likely not suitable bTB biomarkers. However, PPD-B-stimulated AGP can discriminate between bTB_PCR-P_ and bTB_PCR-N_ with a target specificity of 95% but a sensitivity of only 52.38%. Thus, we did not find PPD-B-stimulated AGP to be a suitable biomarker for bTB.

The plasma level of CRP, an activator of the classical complement pathway, increases during an inflammatory state and is widely used as a biomarker for pulmonary infections (35). Previous studies have shown that TB patients display higher CRP levels than healthy controls (36, 37), but in our study, no significant differences in serum CRP levels were observed between M. bovis-infected cattle and NC by iTRAQ analysis. In contrast, CRP levels in the PPD-B-stimulated plasma of bTB_PCR-P_ were significantly higher than those in bTB_PCR-N_ and NC. Similarly, patients with smear- or culture-positive TB exhibited higher CRP levels than smear- or culture-negative patients (38). Considering that host factors (ethnicity, HIV infection) and mycobacterial factors (M. tuberculosis strain type, the site of disease) were strongly associated with the baseline CRP response in TB (39), we assessed the diagnostic efficiency of PPD-B-stimulated CRP using ROC analysis and found that CRP produced a better diagnostic outcome (AUC = 1) than PPD-B-stimulated AGP, unstimulated IL-8, and serum SAA in discriminating between bTB_PCR-P_ and bTB_PCR-N_. Furthermore, clinical testing revealed that, compared to the results of nested PCR, the sensitivity and specificity of the PPD-B-stimulated CRP test were 94% and 97%, respectively, and the agreement between the two tests was also high (κ = 0.9117). Therefore, PPD-B-stimulated CRP displays the potential to differentiate between different stages of M. bovis infection.

### Stages of M. bovis infection can classified by nested PCR.

The serum and PPD-B-stimulated plasma proteomes of bTB_PCR-P_ and bTB_PCR-N_ were compared by iTRAQ analysis, and 74 serum and 201 plasma proteins, respectively, were found to be significantly differentially expressed. These DE proteins showed broad functional distributions by GO and KEGG analyses, and a total of 16 DE serum proteins and 27 DE plasma proteins were found to be involved in the complement and coagulation cascades. Significant differences in serum SAA, serum IL-8, plasma AGP, and plasma CRP levels between bTB_PCR-P_ and bTB_PCR-N_ were also observed by ELISA. A previous study demonstrated that CE-induced or uninduced IL-17A levels were significantly higher in bTB_PCR-N_ than in bTB_PCR-P_ and NC (14). We speculate that bTB_PCR-P_ and bTB_PCR-N_ may be at different stages in the progression of bTB and may produce different immune responses to M. bovis-specific antigens.

### Limitations of this study.

First, our iTRAQ, PRM, and ELISA results did not display complete agreement. The lack of a commercial kit specific for the depletion of high-abundance proteins in bovine samples and the depletion bias produced by the kit used may explain this phenomenon. Second, the iTRAQ, PRM, and ELISA analyses were not conducted at the same time, and thus, some proteins may have degraded during storage. Third, there are few commercial ELISA kits for bovine serum proteins, and some DE proteins, such as CD14 and C1q, displayed strong signals by iTRAQ or PRM analysis but were not validated by ELISA. Finally, it was impossible to slaughter all of the infected cattle used in this study to confirm the stages of bTB progression, including those in the PCR-positive and PCR-negative groups. Thus, we are unable to discern whether our putative biomarkers could be used to differentiate between M. bovis-infected cattle and NC and cattle with active or latent bTB. Therefore, further effort is required to validate these DE proteins as biomarkers for bTB diagnosis.

### Conclusion.

PPD-B-stimulated IL-8 can differentiate between M. bovis-infected cattle and NC, and PPD-B-stimulated CRP can differentiate between bTB_PCR-P_ and bTB_PCR-N_. SAA, AGP, IP-10, and IL-17A are closely related to M. bovis infection but are not preferred markers for bTB diagnosis. The proteome of serum or PPD-B-stimulated plasma in bTB_PCR-P_ was significantly different from that of serum or PPD-B-stimulated plasma in bTB_PCR-N_, indicating that bTB_PCR-P_ and bTB_PCR-N_ are at different stages of bTB progression and display different immune responses to M. bovis-specific antigens.

### Ethics approval.

All animals used in this study were treated with care and with the approval of the Animal Care and Use Committee of the China Institute of Veterinary Drug Control (SYXK 2005-0021).

## Supplementary Material

Supplemental file 1

Supplemental file 2

Supplemental file 3

Supplemental file 4

Supplemental file 5

Supplemental file 6
