# Supplementary material for: Interleukin 8 and Pentaxin (C-Reactive Protein) as Potential New Biomarkers of Bovine Tuberculosis
Source: J Clin Microbiol. 2019 Sep 24;57(10):e00274-19. doi: 10.1128/JCM.00274-19 (PMC6760949; doi:10.1128/JCM.00274-19)
Supplement: Supplemental file 1 [file JCM.00274-19-s0001.pdf]

Supplemental Figures

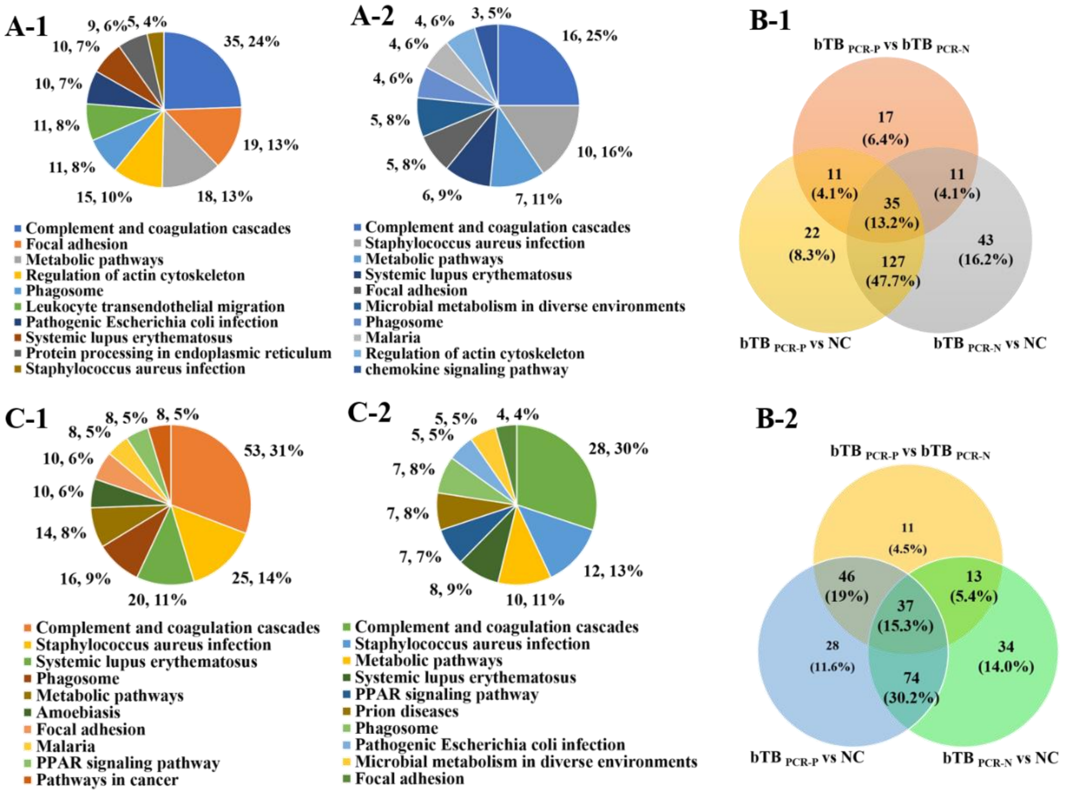

Figure S1. KEGG analysis and Venn analysis of DE serum and plasma proteins.

KEGG analysis: (**A-1**), Serum proteins DE between *M. bovis*-infected cattle and NC; (**A-2**), Serum proteins DE between bTB_{PCR-P} and bTB_{PCR-N} cattle; (**C-1**), PPD-B–stimulated plasma proteins DE between *M. bovis*-infected cattle and NC; (**C-2**), PPD-B–stimulated plasma proteins DE between bTB_{PCR-P} and bTB_{PCR-N} cattle. Venn analysis: (**B-1**), Comparison of DE serum proteins (bTB_{PCR-P} vs. NC, bTB_{PCR-N} vs. NC, and bTB_{PCR-P} vs. bTB_{PCR-N}); (**B-2**), Comparison of DE proteins in PPD-B–stimulated plasma (bTB_{PCR-P} vs. NC, bTB_{PCR-N} vs. NC, and bTB_{PCR-P} vs. bTB_{PCR-N}).

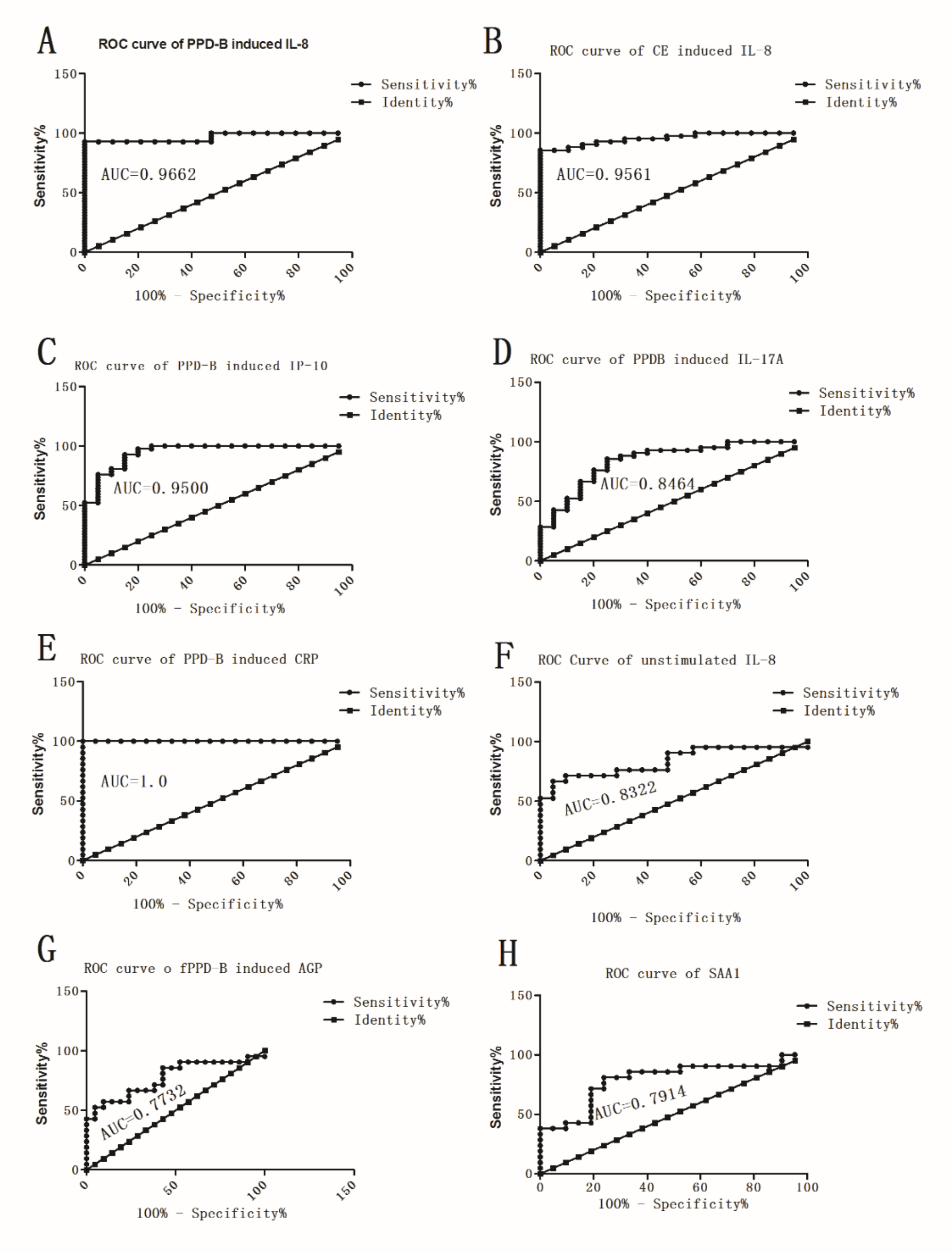

Figure S2. ROC curves.

(A) PPD-B–stimulated IL-8. **(B)** CE-stimulated IL-8. **(C)** PPD-B–stimulated IP-10. **(D)** PPD-B–stimulated IL-17A. **(E)** PPD-B–stimulated CRP. **(F)** Unstimulated IL-8. **(G)** PPD-B–stimulated AGP. **(H)** Serum SAA. The cutoff values for PPD-B–stimulated IL-8, IP-10, or IL-17A ELISAs were obtained using 19 healthy cattle (from a TB-free herd, negative by TST, CET-ST, IGRA, CE-based IGRA, and PCR analysis) and 42 *M. bovis*-

infected cattle (positive by TST, CET-ST, IGRA, and CE-based IGRA). The cutoff values of PPD-B–stimulated CRP, AGP, unstimulated IL-8, and serum SAA ELISAs were obtained using 21 $bTB_{PCR\text{-}P}$ cattle (positive by TST, CET-ST, IGRA, CE-based IGRA, and PCR analysis), and 21 $bTB_{PCR\text{-}N}$ (positive by TST, CET-ST, IGRA, and CE-based IGRA, but negative by PCR analysis). [a] CI. Confidence interval.
